# Incidence, risk factors for metabolic syndrome and health systems capacity for its management amongst people living with HIV, Accra-Ghana: A study protocol

**DOI:** 10.1371/journal.pone.0312446

**Published:** 2024-11-05

**Authors:** Magdalene Akos Odikro, Kwasi Torpey, Margaret Lartey, Peter Puplampu, Elijah Painstil, Ernest Kenu

**Affiliations:** 1 Department of Epidemiology and Disease Control, School of Public Health, University of Ghana, Accra, Ghana; 2 Department of Population, Family and Reproductive Health, School of Public Health, University of Ghana, Accra, Ghana; 3 Department of Medicine & Therapeutics, University of Ghana Medical School, Accra, Ghana; 4 Department of Medicine, Korle Bu Teaching Hospital, Accra, Ghana; 5 Department of Pediatrics, Boston University Chobanian & Avedisian School of Medicine, Boston, MA, United States of America; Ruedi Luethy Foundation - Zimbabwe, ZIMBABWE

## Abstract

**Background:**

Metabolic syndrome (MetS) refers to the clustering of three or more metabolic disorders including high blood pressure, glucose impairment, abdominal obesity, high triglycerides, and low high-density lipoproteins. MetS is increasingly being considered an epidemic among People Living With HIV (PLWH) with reports of association between HIV infection and/or antiretroviral therapy (ART) usage and development of MetS. MetS predisposes PLWH to the development of cardiovascular, kidney diseases and diabetes, decreases the quality of life, and burdens the health system. This study aims to establish the incidence, time to development and risk factors for development of MetS and it’s components, and to assess the capacity of the health system to manage MetS and it’s components among ART naive PLWH in Accra, Ghana.

**Methods:**

We will conduct a mixed methods study with quantitative and qualitative data collection. Our prospective cohort study would enroll adults of 18 years and above with none or less than three MetS components at baseline and follow them up at six months and one year. Demographic, lifestyle data, anthropometric, and laboratory data will be collected using an adapted WHO Steps Survey questionnaire. The WHO Service Availability and Readiness Questionnaire (SARA) will be adapted to collect information on capacity across the six WHO building blocks. Key informant interviews will be conducted with HIV coordinators at the national, regional, and facility levels. In-depth interviews will be conducted with PLWH from the cohort who develop MetS or MetS components during their follow-up. Data will be analysed using proportions, Kaplan Mier time to event analysis, fitting of Cox proportional hazard regression models for risk factors, and generation of themes from qualitative data.

**Expected outcome:**

This study will generate data on the incidence, time to development, risk factors for MetS and MetS components development, and health systems capacity for MetS management among PLWH. Findings would inform revisions to the guidelines and policies for HIV care in Ghana, Africa, and beyond, ultimately improving MetS prevention and management among the vulnerable population of PLWH.

## Background

Globally, over 39 million eople were living with HIV (PLWH) and 1.3 million new HIV infections were recorded in 2022 [1]. The burden of HIV is skewed toward sub-Saharan Africa (SSA) with close to two-thirds of the burden of existing and new HIV infections recorded in the region [1]. The number of new HIV infections and HIV-related mortalities has been on a steady decline with the advancements in the management of HIV. One major advancement that has contributed to the decline in HIV morbidity and mortality is the discovery and use of Anti-Retroviral Therapy (ART). With the advent of ART, new HIV infections have reduced by 59% since the peak of the HIV pandemic in 1997 and the life expectancy of PLWH continue to approach that of HIV-uninfected individuals [1–3].

Increased life expectancy, effects of aging, HIV disease itself, and the use of ART have led to an increase in the development of various non-communicable and cardio-metabolic disorders among PLWH [4–7]. These metabolic disorders include hypertension due to arterial stiffening, glucose impairment (diabetes), weight gain, and blood lipid levels that are too high or too low (dyslipidemia) [8–10]. The clustering of three or more of these identified cardio-metabolic disorders is termed Metabolic Syndrome (MetS) [11]. MetS is more prevalent among PLWH compared to HIV-uninfected populations [10]. Globally, the prevalence of MetS among PLWH ranges from 11% to 48% with higher burdens in Sub-Saharan Africa [10, 12, 13]. In Ghana, MetS prevalence is reported to range between 24% to 48% among PLWH using the WHO and National Cholesterol Education Program (NCEP) criteria respectively [14–18].

Previous research has established a complex pathway between HIV infection, ART, and the development of MetS. MetS predisposes PLWH to the development of cardiovascular and kidney diseases, Type 2 diabetes, and some cancers [10, 19, 20]. The syndrome also decreases quality of life and is strongly associated with increased morbidity and all-cause mortality among PLWH [21]. The increase in morbidity and mortality due to MetS and its components burdens the already fragile health system through increased socioeconomic costs to individuals, families, and the health system [22]. With established differences in genetic, cultural, and environmental factors, it is essential to conduct studies specific to the Ghanaian population to better understand the incidence, time to development, and risk factors associated with MetS development among PLWH. It is also unclear whether the health system in Ghana can appropriately manage this growing epidemic of MetS among PLWH. This prospective study will therefore establish the incidence, time to development, and risk factors for the development of MetS and MetS components among ART naive PLWHs in Ghana. Additionally, a comprehensive assessment of the capacity of the health system for management of MetS among PLWHs will be conducted.

## Methods

### Study setting

The study will be conducted in Ghana, West Africa. Ghana shares boundaries with Côte d’Ivoire, Burkina Faso, and Togo to the west, north, and east respectively. The current estimated population of Ghana is 30.8 million. Ghana is divided into 16 regions. Our study will be conducted in the Greater Accra region of Ghana (GAR). Greater Accra is the capital city of Ghana with the smallest land surface area of about 3,245 square kilometers (14% total land area of Ghana). GAR is the second most populated region inhabiting about 16% of the country’s total population and has the second highest HIV prevalence among the 16 regions.

### Aim and study design

Two main approaches will be utilized to determine the incidence of MetS and MetS components and the capacity of the health system for the management of MetS among PLWH. A prospective cohort study design will be employed to determine the incidence of MetS, time to development, and risk factors. Secondly, a cross-sectional assessment of health system capacity using mixed methods of data collection across selected facilities will be conducted.

### Approach one: Cohort study

#### Study sites

The cohort study will be conducted in four randomly selected high-burden HIV facilities in Greater Accra namely the Tema General Hospital, Korle-Bu Teaching Hospital, Greater Accra Regional Hospital, and Ga West Municipal Hospital with HIV positivity rates of 23.4%, 9.1%, 8.7%, and 9.2% respectively in 2021 [23]. These facilities were randomly selected from the top seven high-volume HIV facilities using data from the National AIDS Control Programme for 2021.

#### Study population

The study population for the cohort study will include all newly enrolled PLWHs receiving care from the four study facilities. All adults 18 years and above newly diagnosed with HIV without a diagnosis of MetS (have none or less than three MetS components) will be eligible for inclusion in the cohort. Although previous incident studies have focused on middle aged and older participants, the increasing prevalence of MetS among adolescents and young adults informed the use of a younger cohort in our study. We will exclude PLWHs who are bedridden, pregnant women, and individuals diagnosed with cancer in order not to inconvenience them with the study processes.

#### Sample size

The sample size was determined using the UCSF sample size calculator Available at [https://www.sample-size.net/]. This sample size calculation uses assumption of normal distribution with it’s continuity correction to approximate the binomial distribution. We estimated that with 250 study participants, we will be able to detect 12-month cumulative incidence (risk) of MetS of 24.4% [from a reference of 37 people developing MetS among 150 PLWH during 12 months of follow up from a recent unpublished follow up study conducted in Ghana] with surrounding 95% Confidence Interval of (19.2, 30.2). This means that the sample size of 250 will give us an 11% width of the confidence interval (precision) and we will be 95% Confident in our estimate. Considering effect size, estimating that 62 people will engage in regular exercise compared to 188 people not engaged in regular exercise, we will have 80% statistical power to detect a between-group difference in risk of 17.8% (among regular exercisers 29% minus among people who do not exercise regularly 11.9%), which translates into a risk ratio of RR = 0.29/0.119 = 2.4. With this, we rounded our sample size to 287 (15% added) to provide a good buffer for any drop out that may be encountered in the cohort.

#### Data collection

**Fig 1:**
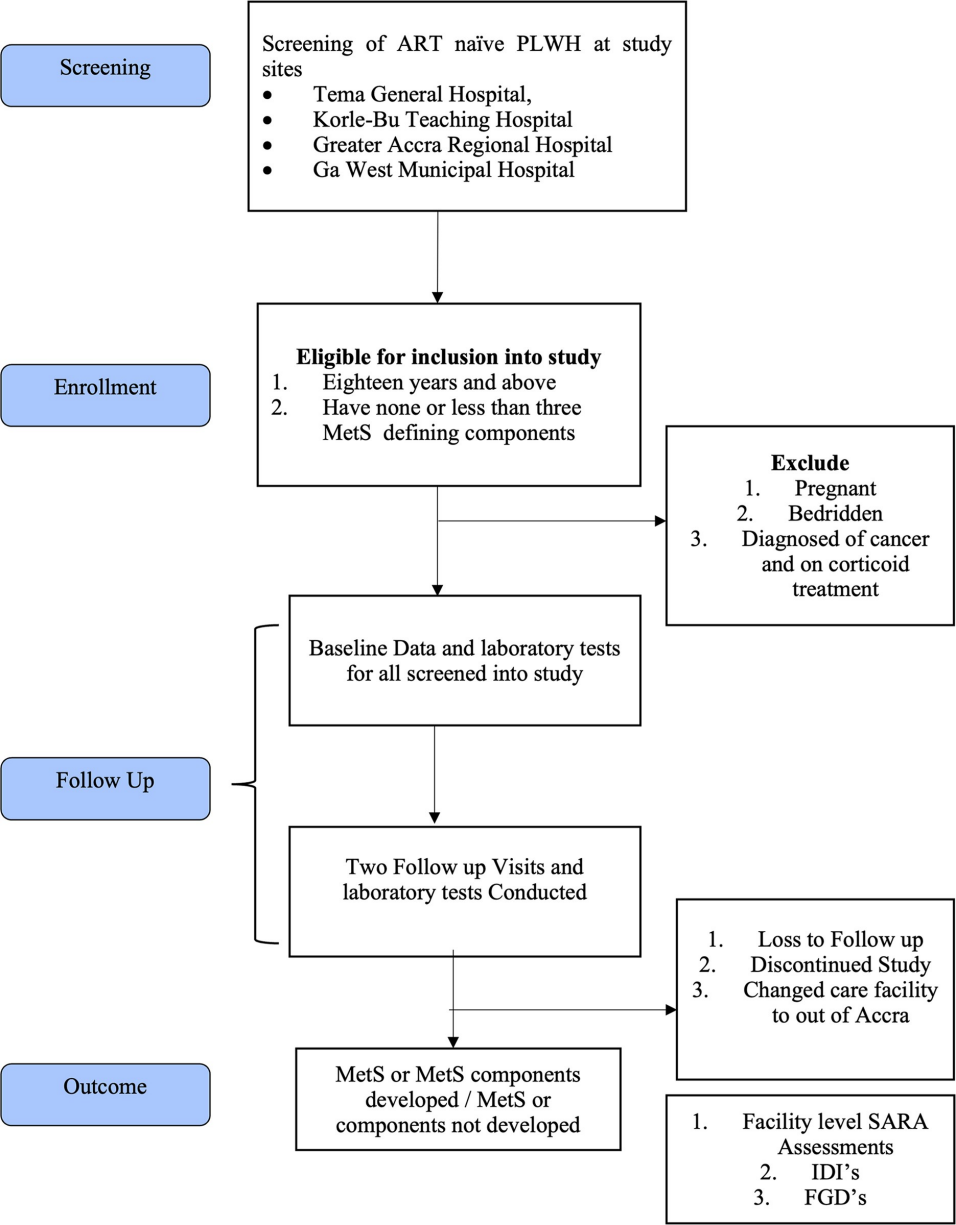
Study processes schema, incidence, risk factors for metabolic syndrome and health systems capacity for its management among newly diagnosed PLWH, Accra.

Across the four recruiting sites, newly diagnosed PLWH who are above 18 years old, and do not have three or more MetS defining outcomes at the point of diagnosis will be enrolled consecutively into the study and followed up for 12 months (Fig 1). All sites will contribute as many new cases concurrently until the studies’ sample size is reached.

A structured questionnaire modified from the WHO Steps survey will be used by trained research assistants to collect data from cohort study participants at baseline, six months, and one year post HIV diagnosis. The WHO steps survey is a standardized structured tool for collecting information on key non-Communicable disease risk factors in countries comprising: Steps 1 –demographics and behavioral measurements covering tobacco use, alcohol use, diet, physical activity, family history of MetS components, HIV-related history, and history related to other comorbidities; Steps 2: physical measurements including blood pressure, height, weight, waist circumference, and hip circumference; and Steps 3: biochemical measurements including blood glucose. Lipids (Total cholesterol, triglycerides, and HDL Cholesterol). Health workers at the sites will be trained to enroll participants based on the study’s eligibility criteria, consent clients and collect the data using the modified WHO steps tool accordingly.

#### Physical measurements for cohort participants

Weight and height will be measured using a standard digital scale and stadiometer (Seca® 763 Germany). Participants will be weighed in the upright position wearing light clothing, and bare feet captured into the nearest 0.5 kilograms. Height will be measured with the participant standing upright without shoes to the nearest 0.1 centimeters.

Right arm systolic blood pressure and diastolic blood pressure will be measured using an automatic oscillometric device (specifically Omron M3 comfort automatic upper arm blood pressure monitor) by trained nurses. Before measurement, participants will relax for 5 minutes. Measurements will be taken with the participant’s feet on the floor and arms supported at heart level. Three measurements will be taken for each participant, two minutes apart and the average reading will be considered the blood pressure reading for the day.

Waist circumference will be measured using a flexible inelastic tape measure of the TBW® brand that has a graduation interval of 0.1 cm to the nearest 0.1 cm. Measurements will be taken with participants standing horizontally at the natural indentation at the end of a normal expiration with participants.

Hip circumference will be measured using a flexible inelastic tape measure of the TBW® brand that has a graduation interval of 0.1 cm at the greatest horizontal circumference below the iliac crest at the level of the widest portion of the buttocks (greater trochanter).

#### Biochemical measurements (Blood sample collection, transportation and analysis)

Participants will be informed to fast overnight for 8 to 10 hours before collection of blood samples for laboratory analysis.

Fasting blood glucose will be measured on-site with calibrated SD CodeFree™ glucometer using capillary blood.

Approximately 5 mls of venous blood will be collected from each participant’s antecubital fossa for analysis. Blood samples will be left undisturbed for 30 mins after which the sample will be spun using a centrifuge and blood serum separated for storage. Frozen serum samples will be transported from each facility using a cold chain (between temperatures of 2°C to 8°C) to a central laboratory for analysis of samples. Fasting blood sugar and lipid profile—high-density lipoprotein cholesterol [HDLc], low-density lipoprotein cholesterol [LDLc], total cholesterol, and triglycerides—analysis will be conducted on the blood samples. Fasting blood glucose and blood sample collection will be repeated at six months and one-year follow-up.

*Follow-up*. Cohort participants will be followed up for 12 months at three intervals; baseline, 6 months, and 12 months. At six months and one year, a simplified version of the questionnaire will be used. Laboratory analysis will be repeated at six months and one year.

### Data processing and analysis

Cohort data will be cleaned and managed using Excel and Stata version 14.1. All interview and laboratory data will be downloaded from Kobo collect, cleaned, and merged. The datasets for the three-time points will then be appended for final analysis. Proportions will be determined for sociodemographic characteristics and reported. The main study outcome of Metabolic syndrome (MetS) will be defined using the Joint Consensus definition that combines the International Diabetes Federation (IDF) and the National Cholesterol Education Program Adult Treatment Panel III (ATP III) definitions as having any three of the following [24]; Elevated waist circumference of >80 cm in women and >94 cm in men (adjusted for the African population), Triglycerides 150mg/dl (3.88mmol/l) or greater or on treatment for dyslipidemia, HDL-cholesterol<40mg/dl (1.03mmol/l) in men and < 50mg/dl (1.29mmol/l) in women or on treatment for dyslipidemia, BP 130/85mmHg or greater or on treatment for hypertension, fasting glucose 100mg/dl (5.6mmol/l) or greater or on treatment for diabetes.

Incidence density will be determined using person time analysis; (Number of new cases that developed MetS or a specific MetS component during study period)/ (Total Person Time at Risk During Study Period). To estimate the time to development of MetS or MetS component, the dataset will be set as survival data with MetS/ specific MetS component as the outcome for analysis of the time to development of MetS. Log rank test will be used to test the statistical significance of the differences in the mean survival time (time to recovery) among different groups and p-values will be reported in a table format. Comparative Kaplan Mier graphs will be drawn for secondary outcomes with high incidence and characteristics with significant differences in their survival times.

We will fit a multivariable Cox proportional hazards regression model to determine the risk factors. At the bivariate level, we will compute associations between independent exposure variables and the development of MetS and MetS component. Crude hazard ratios and their 95% confidence interval will be reported and results with p-values <0.05 will be considered significant at bivariate level. To control for confounding, variables associated with MetS or MetS component development with a significance of 0.2 and other important variables as shown from the literature will be included in building the multivariate model.

### Approach two: Cross-sectional study

#### Study setting

Using data from the National AIDS Control Program, the facilities with the highest reported number of diagnosed HIV-positive patients for 2021 in Accra will be selected for the health facility assessment. A sample of all facilities that diagnosed at least 200 new HIV cases in 2021 in Accra constituting twelve facilities will be included for the cross-sectional facility assessment (Table 1).

**Table 1:** Facilities for health system assessment, MetS among PLHIV, 2022.

No	District	Sub-District	Facility Name	Number Tested for HIV (Population)	Number Tested HIV Positive (Population)
1.	Ashaiman	Mantseman	Trinity Community Hospital	2722	1083
2.	Tema	Tema South	Tema General Hospital*	2405	564
3.	Accra Metro	Ablekuma (Accra Metro)	Korle-bu Teaching Hospital*	4976	455
4.	Weija-Gbawe	Weija	Weija-Gbawe Municipal Hospital	3525	380
5.	Korle-Klottey	North Ridge	Ridge Regional Hospital*	4083	356
6.	Ga West	Amasaman	Ga West Municipal Hospital*	3188	294
7.	Ledzokuku	Teshie North	LEKMA Hospital	4113	290
8.	Ashaiman	Tsinai-agber	Ashaiman Polyclinic	1907	285
9.	Ayawaso East	Kanda	37 Military Hospital	3060	273
10.	Ga East	Haatso	International Health Care Centre	814	254
11.	Korle-Klottey	Adabraka	Adabraka Polyclinic	1820	203
12.	Ga South	Amanfro	Amanfro Polyclinic	457	203

*Sites for recruitment of cohort participants

#### Study population

Key informants from the National AIDS Control Program and healthcare workers from the region and selected study facilities will be part of the study population for the cross-sectional assessment. For the health systems capacity assessment, we will include all facilities in Greater Accra that recorded at least 200 positive HIV cases in 2021.

#### Data collection

Health systems capacity will be assessed at the 12 facilities and Key Informant Interviews and In-Depth Interviews (IDIs) conducted with selected national, regional, and facility heads and PLWH from the cohort who develop MetS or MetS component (Fig 1). The WHO Service Availability and Readiness Assessment (SARA) tool would be modified to assess the health system’s capacity for management of MetS among PLWH. The SARA tool is a standard assessment tool to assess the capacity of health facilities to provide health services including essential medicines, technology, and human resources [25]. This tool has been used in Ghana and other sub-Saharan countries [26, 27]. The adapted version to be used in this study will collect information on facility demographics, source of information on MetS prevention at the facility, MetS management, availability of guidelines and training of personnel in MetS management, and the availability of essential medications for the management of MetS components. The assessment will cover the six WHO health system building blocks ie; service delivery, health workforce, access to essential medicines, financing, health information, and governance building blocks of the health system. In each health facility, trained data collectors will receive permission and use the tool to collect information on service readiness using a combination of observation and interviews with key informants and verification or reported availability and functionality of essential equipment and supplies on-site. Each facility will be visited once for this assessment.

A developed, and pre-tested semi-structured interview guide will be used to interview key informants on the management of MetS among PLWH at the national, regional, and facility levels. All key informants will be interviewed on-site at their places of work during work hours. Interviews will be between 45 minutes to an hour and will be conducted by trained research assistants. Key informant interviews will be recorded.

In-depth interviews will be conducted with PLWHs who develop MetS at the end of the cohort study. A self-developed semi-structured interview guide will be used for this interview. A total of 10–12 PLWHs per study site will be included in the in-depth interviews.

#### Data processing and analysis

Categorical variables from the SARA assessment will be summarized as frequencies and proportions with their corresponding 95% Confidence Intervals (CIs). Continuous variables will be summarized using means and medians as appropriate. Each variable will be assigned a value of 1 if the service or item for offering the service is available and 0 if it is unavailable.

The percentage of facilities reporting specific services offered and their readiness to provide the service will be reported overall and then by facility level, and urban-rural location of the facility. For this study, the six WHO health systems building blocks will be the domains considered for general service readiness including service delivery (basic amenities), health workforce, access to essential medicines, financing, health information, and governance. For every domain, physical presence and current functionality will be established by data collectors on site. For each of the six domains, the score will be generated based on availability of the items and an overall readiness composite score will be generated as the unweighted mean of the domains.

Key informant interviews and in-depth interviews will be transcribed verbatim and analysed using NVivo. Various themes, sub-themes, and corresponding codes will be generated from the qualitative data. The findings from the qualitative data will be used to support the proportions generated from the quantitative data.

### Quality assurance

All data collection tools will be pre-tested after receipt of ethical clearance. The pre-test will be conducted in a health facility in Accra with at least 20 records entered for the adapted WHO steps questionnaire. During the pre-test, the adapted WHO Steps questionnaire will be validated. Training will be organized for two days for research personnel covering survey methods and anthropometry to ensure quality data is collected. They will be recertified semi-annually in anthropometry to ensure high reliability of anthropometry measurements. All study measurement instruments will be calibrated by the Ghana Standards Authority before the study commences.

For laboratory measurements, certification of facility-based laboratory personnel to be engaged for the study will be confirmed. Additionally, laboratory personnel will be trained, and periodic quality assessments will be conducted on selected samples to ensure the correctness of laboratory findings. Data for the cohort study and facility SARA assessments will be entered using an electronic database (RedCap) with preset controls to prevent missing data. For qualitative data, selected transcripts will be back-translated to check the quality of the transcriptions. Quality control will be adhered to and monitored at each stage of data collection, entry, analysis, and report writing.

### Ethics approval and consent to participate

Approval for this study has been received from the Ghana Health Service Ethical Review Committee and the Korle Bu Teaching Hospital Institutional Review Board (KBTH IRB) with approval numbers GHSERC: 019/11/22 and KBTH-IRB 000137/2022 respectively (S1, S2). We will also obtain permission from Regional Health Directorates and participating Health facilities before starting the study. Informed consent will be obtained from participants of the study without any form of coercion and participants will be free to drop out of the study at any point. All responses from respondents will be treated confidentially and used solely for this study. Only the researchers will have access to the collected data/information obtained from participant’s records. We will also make sure that the data that will be gathered are saved and well-encrypted under a password to prevent unauthorized access.

### Status and timeline of study

As at ending of May 2024, the cohort study has begun with about one hundred PLWH recruited so far across the four study sites. The cohort study is estimated to run until August 2025. Health facility assessments and qualitative interviews are scheduled to be conducted from July 2024 to October 2025.

## Discussion

As the life expectancy of PLWHs improves, non-communicable diseases including MetS are becoming more common in this population [10]. This has implications for clinical care and the health system. Newly diagnosed cases of HIV present a unique opportunity to understand the progression of MetS components among PLWH. This study protocol presents our plans to conduct a follow-up cohort study with mixed methods of data collection to determine the incidence, risk factors, time to development, and health system’s capacity for management of MetS among PLWH in Accra, Ghana.

Previous studies have established that the development of MetS among PLWH results from interaction and interplay of demographic, lifestyle, ART, and HIV-related and health system factors [9, 10, 12, 14, 21, 28]. There is a considerable gap in the literature on the estimated time to development of MetS among PLWHs especially among newly diagnosed PLWHs. The existing incident studies on MetS among PLWH did not elaborate much on the time to development. Our study will add to this knowledge gap.

A review of existing guidelines for management of MetS in Sub-Saharan Africa found that over 85% of countries in SSA did not have specific guidelines in place for comprehensive management of the burden of non-communicable diseases among PLWH [22]. In Ghana, guidelines exist for the comprehensive management of components of MetS among PLWH. These guidelines are captured under the differentiated service delivery guidelines of the National AIDS Control Program [29]. Differentiated Service Delivery guidelines for the management of comorbidities among PLWH encourage a one-stop shop for the management of HIV and other comorbidities [29]. With this concept, PLWH should receive routine health education and screening for blood pressure, diabetes, and other non-communicable disease. When detected, management of the conditions is recommended to be integrated into the same clinic [29]. However, research is needed to establish how these guidelines are being applied in the real-world setting and the challenges preventing their holistic implementation. Possible challenges may be encountered across the six building blocks of the health system ie; Service delivery, Health workforce, Access to essential medicines, Financing, Health information, and Governance [30]. Our study will structure the health systems capacity assessment across these blocks.

There are some limitations to the methods proposed in this protocol. For the cohort study, loss to follow-up could impact our final sample size. Due to the psychological and emotional stress of being newly diagnosed, enrolled participants are likely to change their facility of care provision, not accept their diagnosis eventually, or possibly change their minds about participation after they have fully accepted their diagnosis. We have accounted for the loss to follow up in the final sample size calculated for the study. Additionally, some of our variables such as smoking history, exercise habits, and adherence to medications will be collected through self-reports limiting our findings by recall bias. Interviewers will be trained to use prompts and an enabling environment to reduce the risk of recall bias. This study is also limited by the short duration of follow up for the cohort study (12 months). To counter this, additional analysis focusing on the MetS components will be conducted to highlight the changes that will occur in this ART naive cohort. Our study sites are also limited to a maximum of twelve facilities in the Greater Accra Region so findings will reflect the situation in the Greater Accra Region and may not be generalizable beyond this. Lastly, the assessment of the health system’s capacity will be done cross-sectionally. This will only provide a snapshot of the situation at the time of data collection. However, this data on the health system’s capacity will still be very informative for future planning purposes.

Overall, this study will provide valuable data on the incidence, time to development, risk factors for the development of MetS, and the health system’s management capacity for MetS and MetS components among PLWH in Ghana. Findings will be used to inform appropriate revisions to the consolidated guidelines for HIV care in Ghana, the HIV Differentiated service delivery operational manual, the National HIV/AIDS and STI and Ghana NCD policies, and any policies related to HIV and NCD care in Ghana. Recommendations for revisions will focus on the planning of timely interventions to mitigate the development and progression of metabolic abnormalities and the appropriate allocation of resources for strengthening Ghana’s health system for MetS among PLWH ultimately improving MetS prevention and management and reducing its occurrence among this vulnerable population.

Study findings will be disseminated to the facilities, districts, and regions, presentations at international conferences, and publications in peer-reviewed journals.
